# Voltage-Driven Translocation of DNA through a High Throughput Conical Solid-State Nanopore

**DOI:** 10.1371/journal.pone.0046014

**Published:** 2012-09-24

**Authors:** Quanjun Liu, Hongwen Wu, Lingzhi Wu, Xiao Xie, Jinglin Kong, Xiaofeng Ye, Liping Liu

**Affiliations:** 1 State Key Laboratory of Bioelectronics, Southeast University, Nanjing, China; 2 School of Geography and Biological Information, Nanjing University of Posts and Telecommunications, Nanjing, China; 3 SEU-FEI Nano-pico Center, Key Laboratory of MEMS of ministry of education, Southeast University, Nanjing, China; Northeastern University, United States of America

## Abstract

Nanopores have become an important tool for molecule detection at single molecular level. With the development of fabrication technology, synthesized solid-state membranes are promising candidate substrates in respect of their exceptional robustness and controllable size and shape. Here, a 30–60 (tip-base) nm conical nanopore fabricated in 100 nm thick silicon nitride (Si_3_N_4_) membrane by focused ion beam (FIB) has been employed for the analysis of λ-DNA translocations at different voltage biases from 200 to 450 mV. The distributions of translocation time and current blockage, as well as the events frequencies as a function of voltage are investigated. Similar to previously published work, the presence and configurations of λ-DNA molecules are characterized, also, we find that greater applied voltages markedly increase the events rate, and stretch the coiled λ-DNA molecules into linear form. However, compared to 6–30 nm ultrathin solid-state nanopores, a threshold voltage of 181 mV is found to be necessary to drive DNA molecules through the nanopore due to conical shape and length of the pore. The speed is slowed down ∼5 times, while the capture radius is ∼2 fold larger. The results show that the large nanopore in thick membrane with an improved stability and throughput also has the ability to detect the molecules at a single molecular level, as well as slows down the velocity of molecules passing through the pore. This work will provide more motivations for the development of nanopores as a Multi-functional sensor for a wide range of biopolymers and nano materials.

## Introduction

Over the last several decades, nanopores have been developed into powerful and indispensable devices for the investigation of unlabeled biopolymers at single-molecular level since it was firstly reported by Kasianowicz and co-workers in 1996. [1]–[3] It opens a new possible way to read out the sequence of DNA without amplification and labeling. [4], [5] By controlling the velocity, [6] reducing the thickness [7]–[9] and extending the signal bandwidths, [10] both natural and solid-state nanopores have been primarily directed toward low-cost and high-throughput DNA sequencing applications. [11]–[14] However, with the advantages of controllable pore size and shape,[15]–[17] long-term stability and easy integration into detection devices, [17], [18] many valuable translocation results about different samples passing through various solid-state nanopores of suitable size have been reported, which shows that the solid-state nanopores can serve as a Multi-functional sensor for detection and characterization of proteins,[19]–[21] DNA/protein [22], [23] or DNA/ligand complexes,[24]–[26] nano-materials/nanoparticles. [27]–[29].

As a high throughput and label-free sensing tool, nanopores in the range of sub-5 nm and sub-10 nm can unfold and thread DNA molecules into linear fashion,[30]–[32] and offer optimal resolution for the spatial information of DNA molecules at nanometer scale. On the other hand, it is associated with a free-energy barrier for DNA capture, [32], [33] small capture radius, [32] unfavorable interactions [34], [35] and limited lifetime, which severely limit the overall throughput of the method. So, in order to reduce these negative impacts, several or more nanopores with approximate size are needed to achieve enough amount of data. Alternatively, with the advantages of weak DNA–pore interaction and longtime stability, larger nanopores in size of tens to hundreds nanometers are considered for the current DNA analysis with specific characteristics and sensing abilities,[36]–[38] which can ensure free passage of the molecules, suppress the conformational changes of long DNA inside pores and effectively provide a high resolution. However, the signal-to-noise ratio of the blockade current will conspicuously deteriorate if the pore is too large compared to the size of molecules. [37], [38] Hence, the choice of nanopores with suitable dimensions is critical for the design of nanopore devices and understanding the physical mechanism of molecules translocating through nanopores.

Herein, we show our results about the translocation of λ-DNA through a conical nanopore with opening diameters of 30 (tip) and 60 nm (base), fabricated in a 100 nm-thick free-standing Si_3_N_4_ membrane by focused ion beam (FIB), which is much larger and longer than the nanopores of sub 5 nm in 6–30 nm ultrathin membranes reported by far. [7], [16], [17], [32] Thus, it is more stable and not easy to be blocked. λ-DNA molecules were driven through the pore by a set of voltage biases, among which a critical voltage of 181 mV was observed to drive DNA molecules through the pore. The distributions of translocation time and current blockage were investigated as a function of voltage. The current blockage increased and the dwell time decreased linearly over voltages from 200 mV to 450 mV. Events with multiple blockade levels were also got. We find the translocation speed of λ-DNA through the pore is ∼5.56 base/*µ*s at 200 mV, slower than ∼25 base/*µ*s as reported before, [39], [40] which shows our pore can slow down the molecular translocation speed. Moreover, events frequencies as a function of voltage and capture radius were studied. Our results show the unique properties of the nanopore with large size in thick membrane and geometrical aspect ratio can provide more information on translocation behaviors of DNA molecules in a new perspective, which can serve as a long lifetime, high throughput, single-molecular biosensor, without the impact of the pore size.

## Materials and Methods

### Materials

λ-DNA (48.5 kbp, ∼300 *µ*g/mL) was purchased from TaKaRa. KCl and TE buffer solution (pH 8.0) were purchased from Fluka. Polydimethylsiloxane (PDMS) was purchased from Dow Corning, which was curing with a mix ratio 1∶10 at 150°C for 15 min. The other chemical reagents used in our experiments were purchased form Sigma. Milli-Q super purified water with a resistance >18 MΩ/cm was used throughout the experiments. All measurements were performed in 1 M KCl salt solution containing 10 mM Tris-HCl and 1 mM EDTA at pH 8.0 at room temperature. Prior to use, all these solutions were filtered with 0.02 µm Anotop filter (Whatman co.).

### Nanopore Fabrication

**Figure 1:**
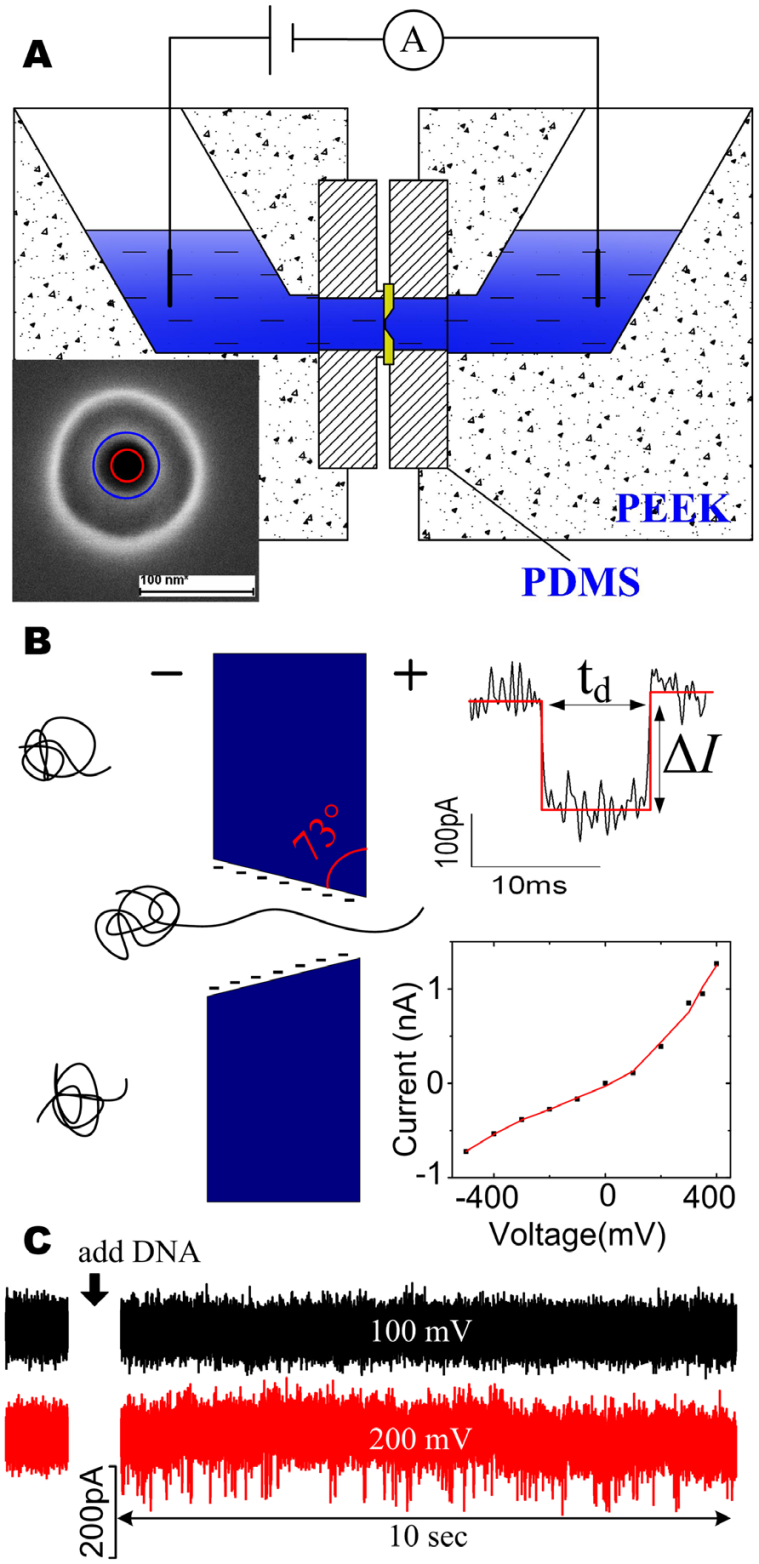
The Schematic illustrations of the microfluidic setup and nanopore detection. A: Schematic illustration of the microfluidic setup. The ionic solution is separated into two isolated reservoirs by the insulating silicon nitride membrane containing a single nanopore. A couple of Ag/AgCl electrodes which connected to the patch clamp amplifier are placed in each of the two reservoirs. Inset is a SEM image of the nanopore fabricated by FIB, with a scale bar of 100 nm. The red circle of 30 nm is used to represent the pore at tip side, while the blue circle of 60 nm is for the pore at base side. **B:** Schematic diagram of single DNA molecule translocating through a nanopore, which results a downward spike in current trace (top inset). The sidewall angle (73°) is calculated and shown in red. Two main parameters: time duration of the blockage (t_d_) and magnitude of the blockage (Δ*I*) are shown for a selected single molecule event. *I*–*V* curve of the conical nanopore is inserted at the bottom, which is smoothed using Savitzky-Golzy method (solid line), showing a typical non-linearity feature. **C:** Current trace recorded at 100 mV and 200 mV, after addition of the λ-DNA molecules, the current shows no spikes when 100 mV is applied (top), whereas a series of events is observed at 200 mV (down). It indicates there is a threshold of electric forces to impel the DNA chain through the pore.

Nanopore used in our study was directly fabricated in free-standing 100 nm thick silicon nitride membrane supported by 300 *µ*m thick silicon wafer (Si 100) using focused ion beam (FIB) sculpting, without further shrinking The thick silicon nitride membrane was deposited by low-pressure chemical vapor deposition (LPCVD) with a 60 *µ*m×60 *µ*m square window exposed for KOH wet etching. Nanopore was drilled in the surface of the membrane by bombarding the surface with Ga^+^ ions using a FEI Strata 201 FIB system at an acceleration potential of 30 kV while the current was measured as 1 pA. The resulting pore was imaged by scanning electron microscopy (SEM) Axiostar plus (ZEISS Axiostar plus), as shown in left inset of Figure 1A and Figure S1. The diameters of both tip side (small pore) and base side (large pore) were analyzed, as the red circle and blue circle shown in the SEM image, where the tip pore is 30 nm and the base pore is 60 nm, resulting a ∼73° sidewall angle (Figure 1B).

### Experimental Setup and Data Acquisition

As shown in Figure 1A, the chip (5×5 mm) carrying a single nanopore was encapsulated in a custom-built PEEK device (Figure S2) with two PDMS films. The two sides of nanopore-containing chip were separated by two PDMS containing one small hole (2 mm) each, which created the fluid connection between nanopore and reservoirs of the PEEK device. A good seal was essential to make sure that the nanopore was the only connection between the two reservoirs which were filled with ionic solutions. Two Ag/AgCl electrodes were inserted into the two reservoirs respectively, and connected to the Axon Multiclamp 700B patch clamp amplifier (Axon Instruments). By applying a bias voltage over the two electrodes, an electric field was established in the area between the electrodes, ideally, in the nanopore and its periphery. The ionic current through the conical nanopore was recorded at a sampling rate of 100 kHz using a 16-bit DAQ card (National Instruments), which was operated with a homemade Labview software. For better signal-to-noise ratio, the signal was filtered at 10 kHz by a low-pass filter embedded in the amplifier. When necessary, a further low-pass digital filter was used, especially at low applied voltages (<300 mV). The whole fluidic device was put in a Faraday cage in order to shield electromagnetic noise. The chip was treated with piranha solution for 30 minutes before the first use, after that, it was kept in water all the time even not use. We found the pore treated in this way have long useable lifetime of more than two weeks. The recorded data was analyzed with the same MatLab and Clampfit (Molecular Devices) routine for translocation events, where events with a dwell time <0.3 ms were ignored. Data was collected over multiple experiments using the same nanopore. The main data used to plot was also put in Table S1.

## Results and Discussion

### Detection of DNA Translocation in Nanopore

The nanopore chip (5×5 mm) was mounted into the fluidic device, as shown in Figure 1A. Then 1 M KCl salt solution was added into two isolated reservoirs. The current-voltage (*I*–*V*) curve was firstly measured and inserted at the bottom of Figure 1B, which is smoothed using Savitzky-Golzy method (solid line), showing a typical non-linearity feature. The conductance was rectified. Both the asymmetric shape of the conical pore and a negatively charged surface of pore wall are considered to be responsible for the current rectification. [41]–[43] Subsequently, λ-DNA was added to the negative reservoir, and was driven through the nanopore by a set of biased voltages from 0 to 450 mV. Transient downward spikes corresponding to the translocation of a single DNA molecule across the pore were detected, as the schematic shown in Figure 1B. Each translocation event is characterized by current blockage (the amplitude: Δ*I*) and translocation time (the width: t_d_), as shown in the top inset of Figure 1B.

**Figure 2:**
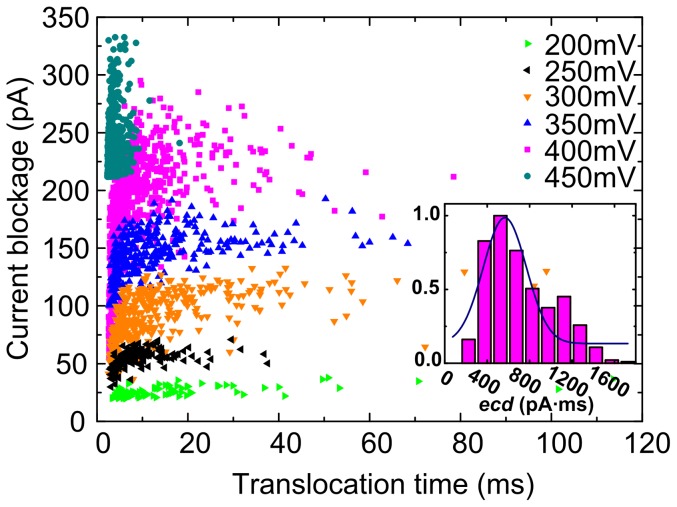
Event scatter plot of current blockage vs translocation time of λ-DNA translocation events as a function of voltage. A typical normalized histogram of 400 mV is inserted on the bottom-right. The experiments were all performed in 1 M KCl solution with 10 mM TrisHCl and 1 mM EDTA at pH of 8.0.

**Figure 3:**
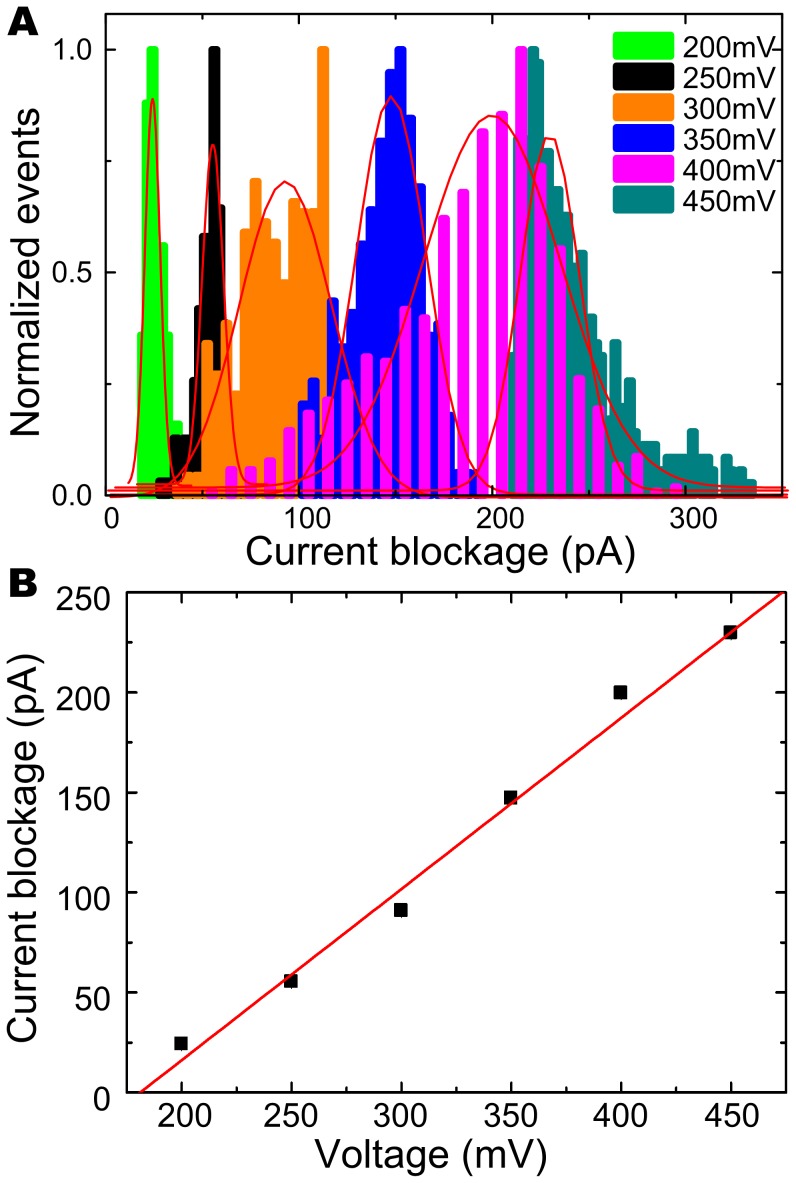
Current blockage histograms as a function of applied voltage. **A:** For comparison all histograms are normalized as shown in the picture, by fitting all the histograms with Gaussian, a increase of the means of the histograms as a function of voltage can be clearly visualized. **B:** The plot of the means of the Gaussian fits of the current blockage histograms as a function of voltage, which is fitted by a line, with a slope of ∼0.85 pA/V and a intercept of 181 mV at voltage axis. It indicates that the current blockage increases with the applied voltage, and a threshold voltage of 181 mV.

Unexpectedly, compared with the open pore current traces, the current blockade events were not observed until a positive voltage of 200 mV was applied, as shown in Figure 1C. Hence, there was a threshold of electric forces to impel the DNA chain through the pore. The scatter plot of translocation events over the parameters t_d_ and Δ*I* at 200 mV, 250 mV, 300 mV, 350 mV, 400 mV, 450 mV is shown in Figure 2. Figure 3A shows the distributions of current blockage, which are fitted by Gaussian. The current blockage increases with the applied voltage. Plot of the mean of the current blockage as a function of voltage in Figure 3B shows the linear relation between current blockage and voltage bias, yielding a slope of ∼0.85 pA/mV and an intercept of −154.92 pA. It indicates a diffusion-limited capture regime above an energy barrier [44] of 181 mV. In contrast with conventional small nanopores, the critical voltage of 181 mV for capturing DNA into the nanopore was higher in our study. [45]–[50] We suppose that the higher threshold voltage is associated with the conical shape and the thickness(100 nm) of the nanopore.

**Figure 4:**
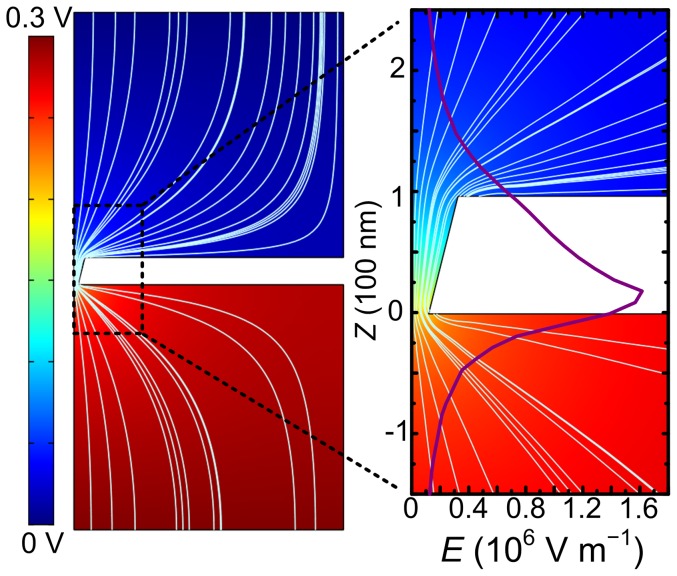
Simulation of the electric potential and field distributions of the nanopore in two dimensions. Left: Color coded potential distribution and electric field lines (white) of the 100 nm thick conical nanopore for a applied voltage of 300 mV. Right: A close-up view of the black rectangle area, integrated with the electric field strength (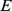) along the center (axis *Z*) of the pore, where the *Z* at the tip side of pore is set as 0. A asymmetric electric field is formed due to the conical shape of the pore, where the electric field strength (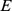) is higher at the tip side than the base side.

As the theoretical model described before, [32], [51] only the DNA coil in bulk that approaches the capture radius of nanopore can be driven through the pore. This process is a thermal diffusion model, in which the DNA’s motion is determined by the electric force acting on it and random thermal forces due to collisions with water molecules. [32], [51] The simulation of electric potential and field distributions of the nanopore in two dimensions was carried using COMSOL Multiphysics v. 3.5a, [19], [27] as shown in Figure 4, where the voltage bias is set as 300 mV. The electric field strength (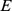) along the center (axis *Z*) of the pore is integrated in the zoomed distribution figure (right), where the Z at the tip side of pore is set as 0. The distribution of the electric field is found to be asymmetric due to the conical shape of the pore, and the electric field strength (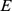) is higher at the tip side than the base side. As the electric force acting on DNA molecule is given by 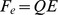, where 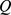 is the charge of DNA, and 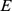 is the electric field strength at the location of the molecule, the electric force acting on the same DNA molecule is larger on the tip side than the base side, with a same distance from the pore mouth. So a higher applied voltage is needed to overcome the thermal forces to drive the DNA to the capture radius on the base side. It indicates that it is more appropriate to add the samples on the tip side of the conical nanopore. [27], [52]

**Figure 5:**
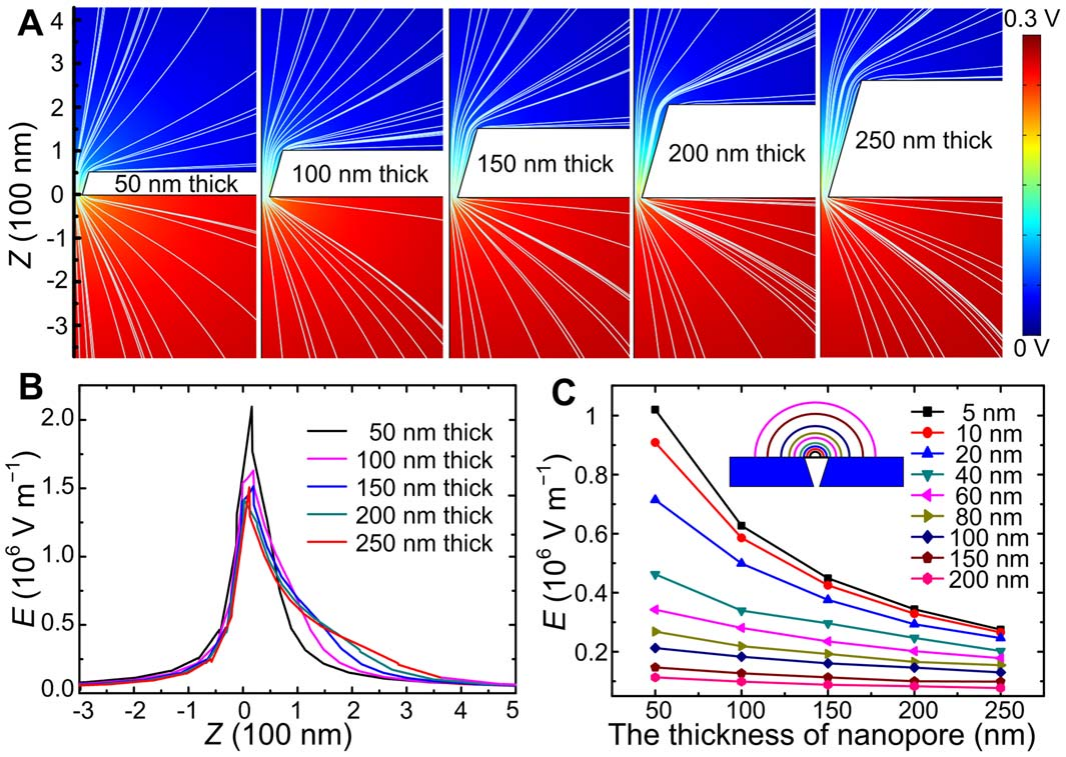
The simulations of the electric field as a function of the conical pore thickness. A: Color coded potential distribution and electric field lines (white) of 50 nm, 100 nm, 150 nm, 200 nm, 250 nm thick conical nanopores with a same tip pore size (30 nm) and sidewall angle (73°), where the applied voltage is 300 mV. For a better comparison, all the *Z* at the tip pore center are set as 0. **B:** The electric field strengths (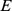) of the five pores as a function of *Z* (−300 nm–500 nm). C: The electric field strengths (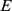) at a set of distances from the base pore as a function of pore thickness. The inset is a schematic illustration of the nine distances from the pore.

Meanwhile, compared to the threshold voltage of ∼600 mV in 10 *µ*m long conical nanopores, [52] the thickness of nanopore may play an important role in dependence of the threshold voltage of the pore. The investigation of electric potential and field distributions in conical nanopores with a different thickness (50 nm, 100 nm, 150 nm, 200 nm, 250 nm) was also made using the COMSOL software, as shown in Figure 5A, where the voltage bias is 300 mV, the tip pore size is 30 nm, and the sidewall angle is 73°. For a better comparison, all the Z at the tip side of the pore are set as 0. Then the corresponding electric field strengths (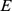) along the pore center (the *Z* axis in Figure 5A) are calculated and plotted together in Figure 5B. It shows that the electric field strength (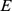) at the pore mouth decreases with the increase of thickness. In order to investigate the correlation of the threshold voltage at base side and the nanopore thickness, we calculated the electric field strength at different distances from the pore centers at the base side (5 nm, 10 nm, 20 nm, 40 nm, 60 nm, 80 nm, 100 nm, 150 nm, 200 nm), as shown in Figure 5C. the inset is a schematic illustration of the nine distances from the pore. The electric field strength at 5–200 nm from the pore at base side decreases with the increase of the thickness, especially at 5–40 nm away from the pore. So the electric force 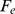 driving the DNA towards to the pore is small in thick nanopore, causing a high threshold voltage to capture the molecules.

In addition, we also notice that the negatively charged surface of the pore wall induces a opposite electroosmotic flow (EOF) and generates a electric field opposite the electrophoretic motion, resulting the pore inhibitory to same charged λ-DNA molecules at low applied voltage (<200 mV), which needs a further investigation. [43], [53]

### Characterization of Translocation Events

**Figure 6:**
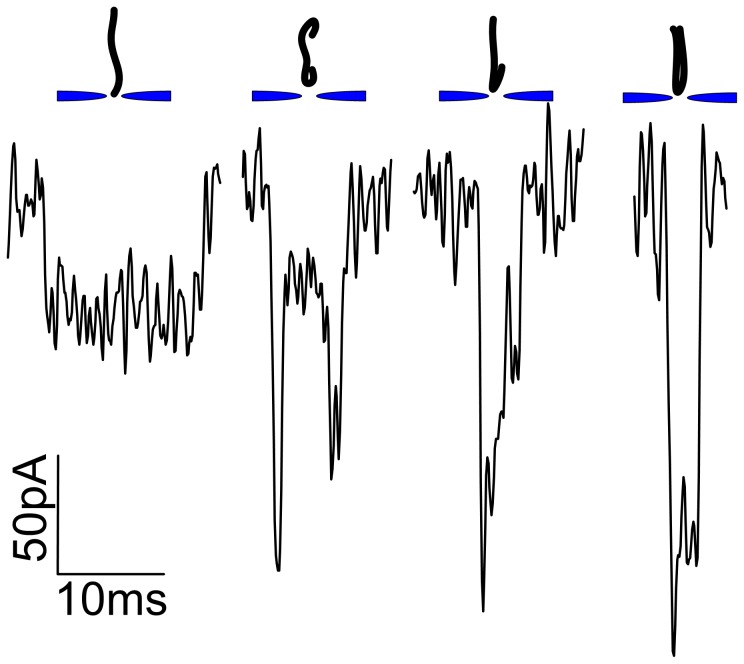
Characteristic signals of translocation events. Four types of typical multiple level translocation events generated by corresponding configurations of DNA molecule, left to right: linear, double local folded, single local folded and fully folded fragments of DNA molecules, respectively.

As mentioned above, events of individual molecular translocations can be characterized by the time duration of the blockage (t_d_) which is the time needed for the molecule to completely translocate through the pore, and the magnitude of the blockage (Δ*I*) which is related to the volume of solution excluded from the pore by the translocating molecule, as indicated in Figure 1B (top inset). A time recording of these current signals during translocations reveals the information about the size and conformation of the passing macromolecule. In our study, a series of multiple blockade levels events were observed, as λ-DNA with various folded configurations transloacating through the pore. A close-up views of the four characteristic events at 250 mV are displayed in Figure 6 as reported before. [39], [40], [54] Unfolded (typical current spike shaped with a broad time and a shallow blockade level attributed to DNA molecule translocates in a linear head-to-tail fashion), partially folded (where the molecule translocates in a randomly local folded forms represented as one (single local folded) or two (double local folded) sharp spikes in Figure 6, with two blockade current levels), and fully folded (The blockade event with a narrower and deeper peak in contrast with the linear form, corresponding to the fully folded molecule translocates through the pore). The result shows that folded λ-DNA molecules pass through the nanopore with shorter dwell time and larger current blockage, while the linear DNA molecules translocate with a longer time and a lower blockade current. In spite of the events with different translocation times and current blockages, these translocation events roughly obey a constant event charge deficit (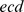), which is defined as the integral area of obstructed ionic current over the duration time resulting from a DNA translocation event, 
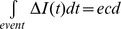
, as reported before. [40], [55], [56] This feature is a signature of translocation of molecules with the same length regardless of their different conformations. Figure 2 shows a scatter plot of Δ*I* versus the time duration (t_d_) of the events at voltage from 200 mV to 450 mV, inserted with a typical histogram of normalized 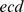 (pA•ms) at 400 mV, which indicates that majority of molecules passed through nanopore in a linear fashion (>60%). Considering the significantly larger dimension of pores compared to the hydrodynamic diameter of DNA, it is possible that long double-strand DNA molecules inherently unfolded and more likely to be captured than the folded ones. Accordingly, extremely small “molecule hugging” pores are not necessary to linearize DNA molecules as they translocate through the nanopore. [39], [55], [56]

### Voltage-dependent Blockade Signals

**Figure 7:**
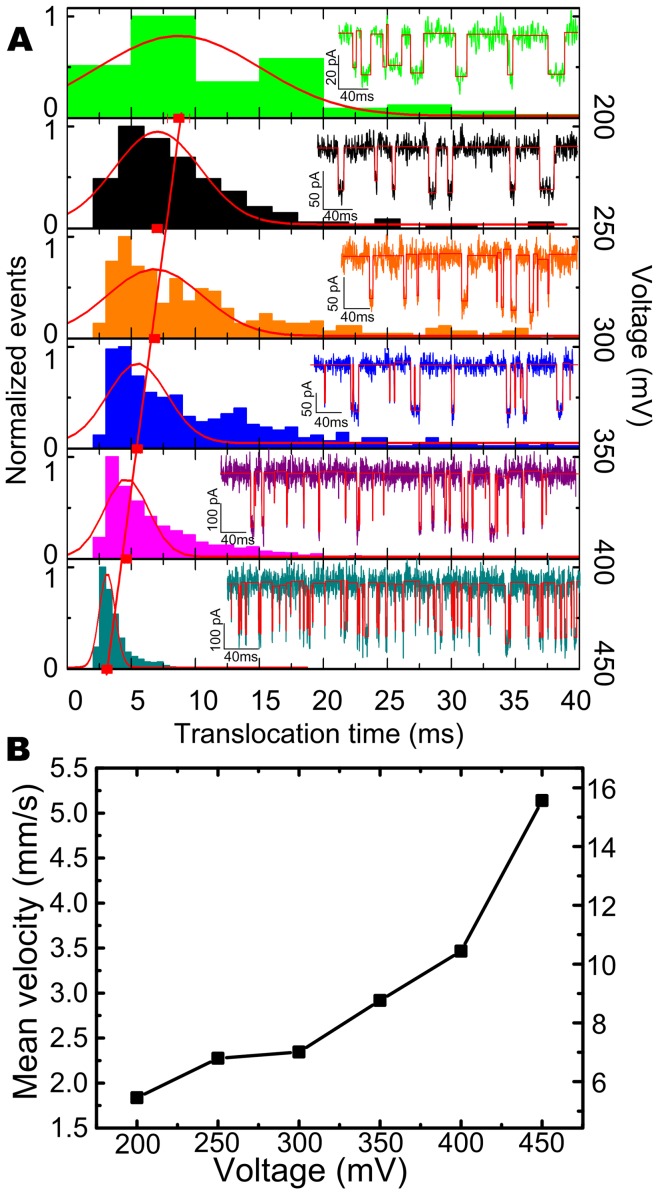
Translocation time histograms and the velocities as a function of applied voltage. A: Normalized histograms of λ-DNA translocation events as a function of voltage are fitted by Gaussian, and aligned for better comparison. A typical current trace with interpretation of events judgment (red square line) of each voltage is insert on the right respectively, from which the events at each voltage can be well distinguished. The means of the Gaussian fits are plotted as a function of voltage (right axis), with a linear fit. **B:** The plot of the velocity (mm/s-left axis, bp/*µ*s-right axis) as a function of voltage.

After having established the ability to discriminate local structures of passing DNA molecule, the voltage effect on the resolution capabilities of translocation signals has been studied using the nanopore. Firstly, the normalized histograms of current blockage at each voltage bias fitted by Gaussian are got and shows in Figure 3A, while the translocation time in Figure 7A. The means of each Gaussian fit of current blockages are plotted as a function of voltage in Figure 3B, [57] which is also shown in Table S1. According to a bigger volume of the pore, a current blockage of 55.409±0.368 pA caused by λ-DNA translocating through the pore at 250 mV is smaller than ∼150 pA at 120 mV in 20–40 nm thick nanopores [39], [40] and 2∼4 nA in 6–15 nm ultrathin nanopores. [7] As mentioned above, the amplitude of the blockage current increases linearly with voltage bias by ∼85 pA per 100 mV, and a threshold voltage of 181 mV is finally got by calculating the intercept of the linear fit at voltage axis. However, the normalized histograms of the translocation time are plotted separately and aligned for better comparison, which are fitted by Gaussian (the red curves), as shown in Figure 7A. For better vision and comparison, the typical current traces at each voltage with interpretation of events judgment (red square line) are inserted on the right of each distribution. The means of the Gaussian fits are also plotted in the same figure (the red line) as a function of voltage (right axis). The linear fit with a slope of −0.023 ms/mV indicates the translocation time strongly decreases with increasing voltage in an reciprocal relationship (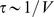). Compared to an exponential function of the translocation time versus voltage (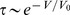) in small pores, this behavior suggests that DNA/pore interactions are attenuated in larger pores. [22], [58]–[60] However, the means of dwell time 8.716±0.863 ms, 7.034±0.302 ms, 6.825±0.281 ms, 5.482±0.238 ms, 4.618±0.127 ms, 3.114±0.409 ms at the biased voltage from 200 mV to 450 mV are longer and than 1–2 ms for the same molecule translocating through the 10–20 nm nanopores in 20–40 nm thick membrane at 120 mV, [39], [46], [59] while the corresponding velocities of λ-DNA (∼16.5 µm) (shown in Figure 7B) translocating at each voltage are ∼5 times slower than that of 20–40 nm thick membrane pores at the same applied voltages. [40] This indicates the pore can slow down the translocation speed of the DNA molecules. We suppose that the velocity of the charged DNA molecules is proportional to electric field strength (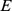) of nanopore via the equation below, as the electrophoretic transport theory described before. [52], [61]

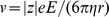
Where 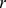 is the radius of the ion, 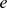 is the electronic charge,and 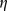 id the viscosity of the solution. With the electric field strength (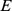) simply written as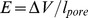, the velocity can be written as



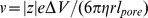
where 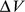
is the voltage applied across the pore, and 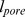
is the length of the pore. As the thickness of the pore in 20–40 nm thick membrane is 1/5∼2/5 of the pore used here in the 100 nm thick membrane, a 2.5∼5 fold slower velocity and longer dwell time are got, which is calculated using 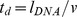, with a constant of 16.5 *µ*m length of λ-DNA. Also we note the high negatively charged surface of the conical pore wall tends to prohibit the insertion and translocation through the pore as shown in Figure 1b, which needs a further investigation.

The throughput of DNA sequencing is mainly determined by the capture probability of molecules into the pore. Generally, the capture process involves two steps. [32], [40] At first, a DNA coil in bulk approaches the pore from purely diffusive to biased motion, driven by the electric field near the pore. Subsequently, DNA searches for an initial conformation and thread into pore mouth by overwhelming a free-energy barrier. When the capture rate is limited by the thermal diffusion, this can be described by Smoluchowski theory for absorption by a hemisphere of radius 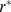. the diffusion-limited capture rate 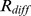 is given by 
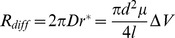
, where *D* is DNA diffusion coefficient, 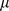 is DNA electrophoretic mobility, 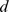 is the pore diameter and 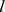 is its length. [32] When DNA capture is governed by an energy barrier, its rate, according to classical Kramers theory, can be written in the form 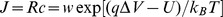, where 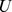 is the height of the threading barrier without any voltage applied and 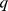 is the effective charge of a DNA end segment. [62].

**Figure 8:**
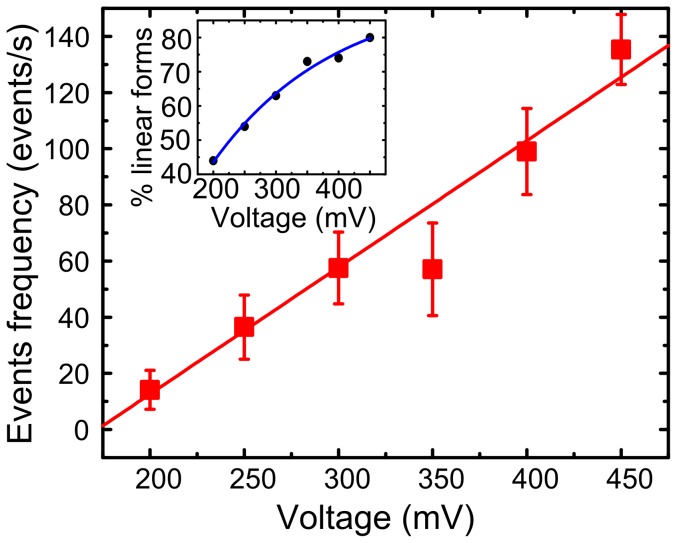
Translocation events frequencies (events per second) versus applied voltage. Translocation events frequency (▪) of λ-DNA (∼300 µg/mL, 1 M KCl TE solution at pH of 8.0) through the pore as a function of voltage, which is fitted by a line (red). The inset is a pot of the percentage of linear form translocation events (•) as a function of voltage. the blue line is a exponential fit.

As recently reported, the threading energy barrier in small pores governs the throughput of nanopore sensing, as observed by an exponential dependence of capture rate on voltage. [22], [32], [58]–[60], [63] To determine the rate-limiting step in larger nanopores, the voltage dependence of the capture rate was also studied in our work. The data of events frequencies are calculated and shown in Table S1. However, we find the blockade events frequency is linearly proportional to the applied voltage, as represented in Figure 8. The plot of the events frequencies as a function of voltage is fitted by a linear function, yielding a slope of 0.45 V^−1^, which indicates the capture process of λ-DNA obeys a thermal-diffusion regime in our experimental conditions. [40], [52] With increase of the biased voltage, an electric field profile is enlarged to a farther distance around the pore mouth, which drives more molecules into the pore. We also note that the events frequencies shown in Figure 8 are ∼2 orders of magnitude larger than previous results reported by Chen. [40] Considering the concentration of λ-DNA we used in our experiments which is ∼60 fold than Chen’s, we also calculate the capture radius using 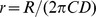, where *R* is the capture rate, 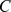 is the molar concentration, and 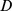 is the diffusion constant assumed as ∼6×10^−9^ cm^2^/s. [64] A 5.98 *µ*m capture radius at 450 mV is got, which is larger than Chen’s 2.7 *µ*m in 15 nm nanopore at 500 mV. It indicates larger pore can enhance the throughput of the nanopore sensing.

Meanwhile, we observed a similar phenomenon that the multiple level of blockade events is gradually diminished and more molecules translocated through the pore in a linear fashion with the increase of the applied voltage, [40] as shown in the inset of Figure 8. As interpreted before, once DNA molecules are captured by the local electric field near it, the electric field within the nanopore then forces the DNA molecules to pass through the pore. This coiled λ-dsDNA must be stretched by the biased forces acting on the different fragments of DNA in and outside the pore due to the nonuniform distributed electric profile. [44], [47], [50], [65] A greater biased voltage produces a stronger electric force. As a result, more molecules are captured by the extended electric field, and the random coil structure at equilibrium is unfolded by the steep electrical forces. Thus the translocation events turned to be a single file manner with the increase of biased voltages.

### Conclusion

The experimental results presented here demonstrate that how a voltage biased solid state nanopore can serve as a high throughput single molecule sensing device, by electrophoretically driven negatively charged λ-DNA through a nanopore at a sets of voltage bias (200–450 mV), Our results show that the nanopore fabricated in silicon nitride membrane by FIB with unique geometrical aspect ratio, larger size (30–60 nm) and long channel (100 nm) not only can be used to characterize the presence and configurations of the translocating molecules, buy also serve as a long lifetime, high throughput single molecule sensor. An external electric field is required to drives the negatively charged DNA through the pore as a threshold voltage of 181 mV is observed. The simulations of the electric field as a function of the conical pore thickness were carried, which shows the threshold voltage is associated with the conical shape and the thickness(100 nm) of the nanopore. As expected, the current blockage increases with applied voltage, while the translocation time decreases. However, due to the thick silicon nitride membrane and the high negatively charged pore wall, we find a ∼5 times slower translocation speed than that of ultrathin (20–40 nm) membrane pores, which is favorable to improve the accuracy of the measurements and probe the dynamics of the translocating biomolecules through the pore. Moreover, the events frequencies of the pore at each applied voltage are analyzed, and the results show the events frequencies increase linearly with voltage at a rate of 45 per 100 mV, which indicates that the pore of larger diameter can capture more molecules than the smaller ones at the same experimental condition.

Overall, the process of DNA transport through nanopores is intriguing for a fundamental perspective of investigating polymer behavior translocating in confined volumes as well as the practical applications of single molecule detectors. In our work, the voltage-driven DNA translocation through larger nanopore has been investigated by an integrated patch-clamp amplifier system. The results show that large nanopore in think membrane, with improved stability and throughput, also has the ability to detect the molecules at single molecular level, as well as slow down the velocity of the molecules passing through the pore. These studies may provide more motivation for the development of nanopores as a Multi-functional sensor for a wide range of biopolymers and nano materials.

## Supporting Information

Figure S1 **The SEM image of the conical nanopore and the analysis of the pore diameters of both tip and base side.**
(DOC)

Figure S2 **The custom-built fluidic PEEK device and nanopore chips.**
(DOC)

Table S1 The main data used to plot.
(DOC)
