# Supplementary material for: Voltage-Driven Translocation of DNA through a High Throughput Conical Solid-State Nanopore
Source: PLoS One. 2012 Sep 24;7(9):e46014. doi: 10.1371/journal.pone.0046014 (PMC3454345; doi:10.1371/journal.pone.0046014)
Supplement: Figure S1 — The SEM image of the conical nanopore and the analysis of the pore diameters of both tip and base side. (DOC) [file pone.0046014.s001.doc]

**Figure S1. The SEM image of the conical nanopore simply fabricated by FIB**


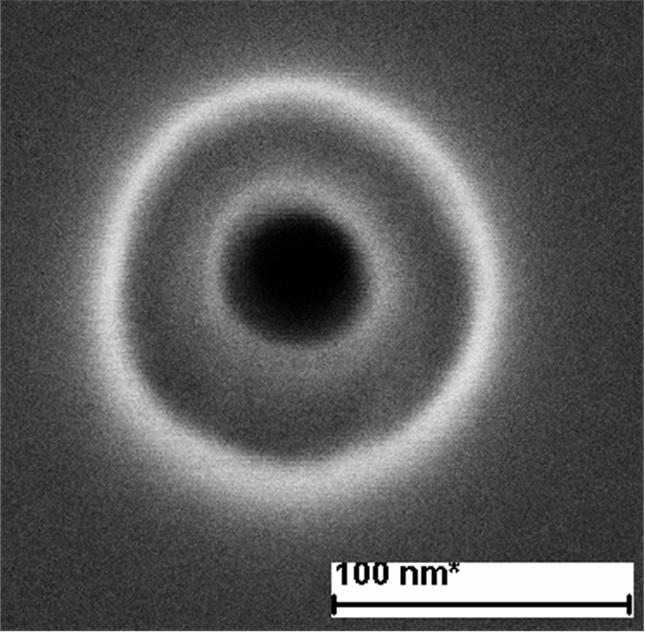

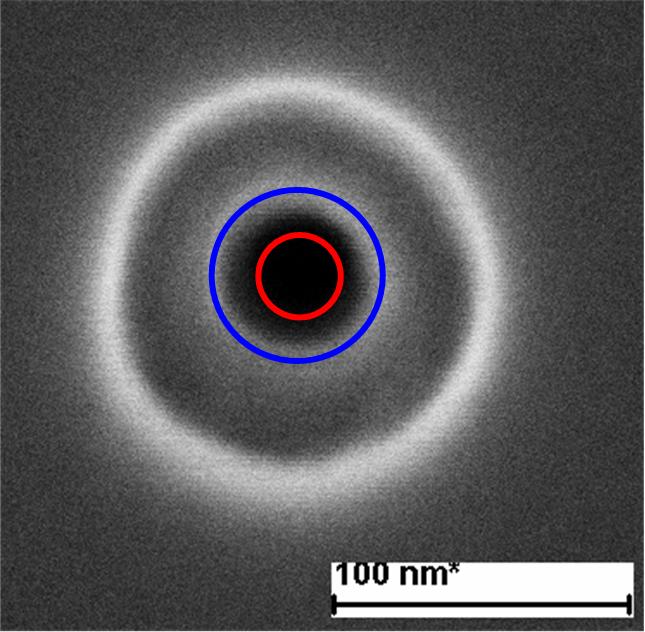


**Figure S1. the analysis of the pore diameters of both tip and base side.**
