# Supplementary material for: Voltage-Driven Translocation of DNA through a High Throughput Conical Solid-State Nanopore
Source: PLoS One. 2012 Sep 24;7(9):e46014. doi: 10.1371/journal.pone.0046014 (PMC3454345; doi:10.1371/journal.pone.0046014)
Supplement: Figure S2 — The custom-built fluidic PEEK device and nanopore chips. (DOC) [file pone.0046014.s002.doc]

**Figure S2. The custom-built fluidic PEEK device and nanopore chips**

The fluidic device is shown in figure 1. Two typical dimensions of the nanopore-containing chip for TEM fabrication (diameter ~ 2.8mm) on the left and FIB fabrication (5mm × 5mm) on the right are in shown in figure 2. Here, the chip (5 × 5 mm) carrying a single nanopore fabricated by FIB was used.


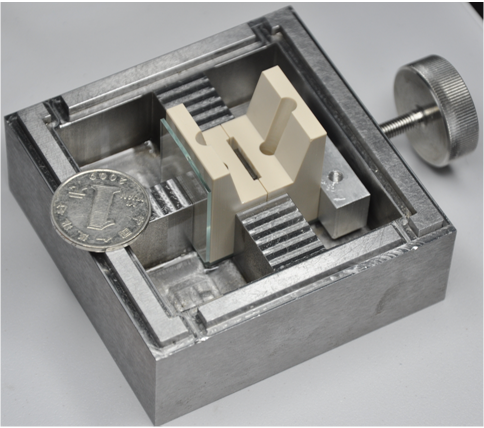

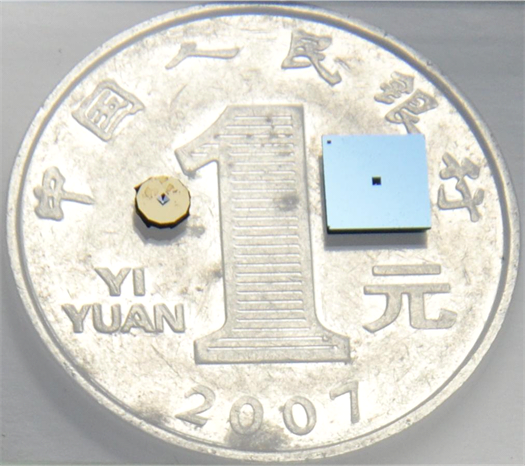


**Figure S2. Left: The custom-built fluidic PEEK device**. Right: Two typical nanopore containing chips. The chip on the right (5 × 5 mm) carrying a single nanopore fabricated by FIB was used in this work.
