# Supplementary material for: Voltage-Driven Translocation of DNA through a High Throughput Conical Solid-State Nanopore
Source: PLoS One. 2012 Sep 24;7(9):e46014. doi: 10.1371/journal.pone.0046014 (PMC3454345; doi:10.1371/journal.pone.0046014)
Supplement: Table S1 — The main data used to plot. (DOC) [file pone.0046014.s003.doc]

**Table S1. The main data used to plot**

the means of the Gaussian fits of the translocation time and current blockage, the velocities, and the events frequencies as a function of voltage are shown in the table.

Table S1: The data used to plot

| Voltage | The mean of Gaussian fit of translocation time | The mean of Gaussian fit of current blockage | Velocity | | Events frequency |
| --- | --- | --- | --- | --- | --- |
| (mV) | (ms) | (pA) | (mm/s) | (bp/*u*s) | (events/s) |
| 200 | 8.716 ± 0.862 | 24.211 ± 0.619 | 1.835 | 5.564 | 14.109 ± 6.936 |
| 250 | 7.034 ± 0.302 | 55.409 ± 0.368 | 2.274 | 6.895 | 36.507 ± 11.424 |
| 300 | 6.825 ± 0.281 | 90.943 ± 1.154 | 2.344 | 7.106 | 57.507 ± 12.770 |
| 350 | 5.482 ± 0.238 | 147.159 ± 0.509 | 2.918 | 8.846 | 57.041 ± 16.483 |
| 400 | 4.618 ± 0.127 | 199.725 ± 1.712 | 3.464 | 10.501 | 99.0281 ± 15.339 |
| 450 | 3.114 ± 0.040 | 229.706 ± 0.598 | 5.137 | 15.573 | 135.343 ± 12.493 |
